# Hydrogen Sulfide Alleviates Postharvest Senescence of Grape by Modulating the Antioxidant Defenses

**DOI:** 10.1155/2016/4715651

**Published:** 2016-08-10

**Authors:** Zhi-Jing Ni, Kang-Di Hu, Chang-Bing Song, Run-Hui Ma, Zhi-Rong Li, Ji-Lian Zheng, Liu-Hui Fu, Zhao-Jun Wei, Hua Zhang

**Affiliations:** ^1^School of Food Science and Engineering, Hefei University of Technology, Hefei 230009, China; ^2^Biological Science and Engineering College, Beifang University of Nationalities, Yinchuan 750021, China

## Abstract

Hydrogen sulfide (H_2_S) has been identified as an important gaseous signal in plants. Here, we investigated the mechanism of H_2_S in alleviating postharvest senescence and rotting of Kyoho grape. Exogenous application of H_2_S released from 1.0 mM NaHS remarkably decreased the rotting and threshing rate of grape berries. H_2_S application also prevented the weight loss in grape clusters and inhibited the decreases in firmness, soluble solids, and titratable acidity in grape pulp during postharvest storage. The data of chlorophyll and carotenoid content suggested the role of H_2_S in preventing chlorophyll breakdown and carotenoid accumulation in both grape rachis and pulp. In comparison to water control, exogenous H_2_S application maintained significantly higher levels of ascorbic acid and flavonoid and total phenolics and reducing sugar and soluble protein in grape pulp. Meanwhile, H_2_S significantly reduced the accumulation of malondialdehyde (MDA), hydrogen peroxide (H_2_O_2_), and superoxide anion (O_2_^∙−^) in grape pulp. Further investigations showed that H_2_S enhanced the activities of antioxidant enzymes ascorbate peroxidase (APX) and catalase (CAT) and decreased those of lipoxygenase (LOX) in both grape peels and pulp. In all, we provided strong evidence that H_2_S effectively alleviated postharvest senescence and rotting of Kyoho grape by modulating antioxidant enzymes and attenuating lipid peroxidation.

## 1. Introduction

Grapes are subject to postharvest senescence during storage, in the syndromes of serious water loss, berry softening, off-flavour occurrence, and decay caused mainly by* Botrytis cinerea*, which reduces the commodity and consumption of grapes [1]. Besides, rachis browning is also an important storage problem of grapes, which greatly affects consumer preference and fruit price [2]. The universal practice to control postharvest decay is to fumigate grapes with SO_2_. Despite the obvious effect of SO_2_ in controlling fungal spreading and postharvest rotting, SO_2_ treatment causes tissue damage to grape berry such as cracks and bleaching and also leads to excessive sulfite residue which may induce allergenic effects [3, 4]. Thus, developing novel technologies to prolong the shelf life of grapes is of great importance to both grape growers and consumers.

Hydrogen sulfide (H_2_S) has been identified as a third multifunctional endogenous gaseous signal after nitric oxide (NO) and carbon monoxide (CO) in animal system [5]. In plants, H_2_S emission has been found in many plant species such as cucumber, squash, pumpkin, soya bean, and cotton [6, 7]. More recently, the metabolism and function of endogenous H_2_S have been clarified through H_2_S-generation defect mutants, revealing its role in plant growth and development [8–10]. Accumulating evidence indicates that H_2_S functions in various processes in plants, including seed germination, root organogenesis, abiotic stress tolerance, photosynthesis, guard cell movement, and postharvest senescence, suggesting that H_2_S acts as an important signaling molecule in plants, as do NO and CO [11–17].

Fruit senescence is usually accompanied by physiological and biochemical changes among which oxidative damage caused by reactive oxygen species (ROS) such as O_2_^∙−^ and H_2_O_2_ are universally observed [18]. Several recent studies found that H_2_S could attenuate oxidative stress by modulating antioxidant enzymes in some postharvest fruits and vegetables including strawberry, mulberry, kiwifruit, and broccoli [19–22]. However, there is no study on whether H_2_S plays a role in delaying the senescence of postharvest grape. In the present research, H_2_S donor sodium hydrosulfide (NaHS) solution was applied to fumigate grapes and the effects of H_2_S signal on grape senescence, the metabolism of natural antioxidants, and ROS and on the activities of antioxidant enzymes were investigated.

## 2. Materials and Methods

### 2.1. Plant Material and Treatments

Clusters of fresh Kyoho grape (*Vitis vinifera* L. ×* V. labrusca* L. cv. Kyoho) were kindly supplied by the orchard of Anhui Academy of Agricultural Sciences, Anhui, China, and grape samples of commercial ripeness, similar bunch size, and no disease and injury were used in this study. Solution of sodium hydrosulfide (NaHS·3H_2_O, Sigma) was used as H_2_S donor. Aqueous solutions of NaHS at different concentrations (150 mL) of 0, 0.20, 0.40, 0.60, 0.80, 1.00, 1.20, 1.40, 1.60, 1.80, 2.00, or 2.20 mM were prepared in sealed containers (volume 3 L) and the solutions were renewed daily. Twelve groups of grape clusters in three replicates (approx. 150 g per replicate) were fumigated with H_2_S in the sealed containers at 25°C and a relative humidity of 85–90%. Grape clusters exposed to H_2_S fumigation were photographed daily, and the rotten and threshing berries of three replicate grapes were recorded. Rotten fruit rate (%) = (the number of rotten berries (berries with mildew or rot) + the number of threshing berries)/total number of berries in a replicate.

### 2.2. Quality Evaluation of Grapes

Grape clusters were fumigated with water or H_2_S released from 1.0 mM NaHS in the sealed containers at 25°C and a relative humidity of 85–90% for 7 days and relative data were analyzed. Browning index of grape rachis was evaluated according to the browning scales as follows: 0, no browning; 1, browning scale less than a quarter of total area of rachis; 2, browning of scales less than 1/2 of total area of rachis; 3, browning of scales less than three-quarters of total area of rachis; and 4, more than 3/4 of total area of rachis. Browning index (BI) was calculated daily by the following formula: BI = ∑(*df*)/*ND*, where *d* is the browning of scales on the grape rachis and *f* is its respective quantity; *N* is the total number of grape rachis examined; and *D* is the highest browning of scales.

For weight loss percentage, the weight of grape clusters was measured before treatment (*a*) and after storage (*b*). The weight loss was calculated as (*a* − *b*)/*a*.

Grape firmness was measured at the equatorial part of individual grape by a 5-mm diameter flat probe with a texture analyzer (Model TA XT plus, SMS). The penetration depth was 5 mm and the crosshead speed was 5 mm·s^−1^. Fruit firmness values were an average of 8 grape berries ± SD (standard deviation).

The total soluble solids (TSS) were determined by measuring the refractive index of the fruit with a hand refractometer (Tongfang Inc., Shanghai, China) according to the method of Jiang et al. [23]. The values were an average of 10 replicates of grapes ± SD.

The titratable acidity of the grape (pooled juice of 15 berries, three replicates per treatment) was measured by titration with 0.1 mM NaOH to pH 8.3. The results were expressed as g·L^−1^ [24].

### 2.3. Determination of Chlorophyll and Carotenoid Contents in Grape Rachis and Pulp

Chlorophyll content of grape was determined using the colorimetric method according to Lichtenthaler and Wellburn [25] with minor modifications. About 5.0 ± 0.05 g of finely chopped grape flesh samples or 2.5 ± 0.02 g of finely chopped grape rachis samples was homogenized using a pestle and mortar on ice and incubated in an Erlenmeyer flask containing 10 mL of 80% acetone as extraction solvent. After extraction in darkness for 24 h at 4°C, the supernatant was measured at 663 and 645 nm, respectively. Chlorophyll and carotenoid contents were calculated with the following equations: Carotenoid = *A*_440_ *V*/*W*; Chla = (12.7*A*_663_ − 2.69*A*_645_)*V*/*W*; Chlb = (22.9*A*_663_ − 4.68*A*_645_)*V*/*W*; and Chl = Chla + Chlb. Chlorophyll and carotenoid contents were expressed as mg·g^−1^ FW.

### 2.4. Determination of Ascorbic Acid, Flavonoid, Total Phenolics, Reducing Sugar, and Soluble Protein in Grape Pulp

Ascorbic acid was determined by the method described by Nath et al. [26]. Grape pulp samples 5.0 ± 0.05 g were ground with 15 mL of 4% oxalic acid. After centrifugation at 5,000 rpm for 15 min at 4°C, the supernatant was adjusted to 25 mL with 2% oxalic acid and titrated with 2,6-dichlorophenol-indophenol to a pink color.

Determination of total phenolics and flavonoid in grape pulp was performed according to the methods of Pirie and Mullins [27] and Zhishen et al. [28], respectively.

Soluble protein and reducing sugar contents in grape pulp were measured according to Bradford [29] and Miller [30], respectively. Grape pulp samples at 5.0 ± 0.05 g were ground with 3 mL of sodium phosphate buffer (pH 7.0, 200 mM), and the homogenate was centrifuged at 10,000 rpm for 30 min at 4°C. Then, the supernatant was collected for the determination of soluble protein and reducing sugar content. For soluble protein, 0.1 mL of supernatant was mixed with 0.9 mL of dH_2_O and 5 mL of Coomassie Brilliant Blue. Absorbance was recorded at 595 nm after 5 min. The results were expressed as *μ*g·g^−1^ FW.

Reducing sugar was measured by the dinitrosalicylic acid method. The supernatant (0.2 mL) was mixed with 1.5 mL of 3,5-dinitrosalicylic acid and 1.8 mL of dH_2_O, and then the mixture was heated at 100°C for 5 min, cooled, and added to 25 mL distilled water. Reducing sugar was determined at 540 nm by a spectrophotometer, and the results were expressed as mg·g^−1^ FW.

### 2.5. Determination of Malondialdehyde (MDA), Hydrogen Peroxide (H_2_O_2_), and Superoxide Anion (O_2_^∙−^) in Grape Pulp

Contents of MDA and H_2_O_2_ and generation of O_2_^∙−^ were determined according to the methods described by Hu et al. [22] with minor modifications. For MDA analysis, grape pulp samples (5.00 ± 0.05 g) were ground in liquid nitrogen and extracted in 3 mL 0.1% trichloroacetic acid (TCA). The homogenate was centrifuged at 12,000 rpm for 20 min and 1.8 mL of the supernatant fraction was mixed with 1.8 mL of 20% TCA containing 0.5% thiobarbituric acid. The mixture was incubated at 100°C for 30 min, cooled, and centrifuged at 12,000 rpm for 10 min. Absorbance was recorded at 532 nm, and the value for nonspecific absorption at 600 nm was subtracted. An extinction coefficient of 155 mM^−1^·cm^−1^ was used to calculate MDA content which was expressed as *μ*mol·g^−1^.

For determination of H_2_O_2_, grape pulp samples (5.00 ± 0.05 g) were ground and extracted in 3 mL cold acetone. The homogenate was centrifuged at 12,000 rpm at 4°C for 30 min and 0.5 mL of the supernatant fraction was mixed with 1.5 mL of CHCl_3_ and CCl_4_ (1 : 3, V/V) mixture. Subsequently, 2.5 mL of distilled water was added and the mixture centrifuged at 12,000 rpm for 1 min and the aqueous phase collected for determination. The reaction system included 1 mL sample, 0.5 mL of buffer (phosphate-buffered saline, 200 mM, pH 7.8), and 20 *μ*L (0.5 unit) of catalase as control or inactive catalase protein (catalase inactivated by heating in boiling water for 5 min). After the mixture was incubated at 37°C for 10 min, 0.5 mL of 200 mM titanium 4-(2-pyridylazo) resorcinol (Ti-PAR) was added. The reaction mixture was incubated at 45°C for another 20 min. Absorbance at 508 nm was measured and H_2_O_2_ content was indicated as *μ*g·g^−1^ FW.

The generation rate of O_2_^∙−^ was determined using hydroxylamine method. Grape pulp samples (5.00 ± 0.05 g) were ground with 3 mL of 50 mM Tris-HCl buffer (pH 7.8) and the homogenate was centrifuged at 12,000 rpm at 4°C for 30 min. The reaction mixture (0.5 mL) contained 50 mM Tris-HCl buffer (pH 7.5), 0.5 mM XTT [sodium, 3-1-(phenylamino-carbonyl)-3, 4-tetrazolium-bis(4-methoxy-6-nitro), and benzenesulfonic acid hydrate], and 50 *μ*L of sample extracts. Corrections were made for the background absorbance in the presence of 50 U of superoxide dismutase (SOD).

### 2.6. Activity Assays of APX, CAT, and LOX in Grape Peels and Pulp

Activities of ascorbate peroxidase (APX, EC 1.11.1.11) and catalase (CAT, EC 1.11.1.6) were determined by the procedures described by García-Limones et al. [31]. Grape pulp samples (5.00 ± 0.05 g) or grape peel samples (2.0 ± 0.02 g) were homogenized with 5 mL of ice-cold sodium phosphate buffer (50 mM, pH 7.5, containing 5 mM beta mercaptoethanol and 1% polyvinyl pyrrolidone). The homogenate was centrifuged at 10,000 rpm (4°C, 20 min), and the supernatant was used for activity measurement.

APX activity was determined by the decrease of ascorbate which was measured in absorbance at 290 nm. The reaction mixture contained 2.5 mL 50 mM K-phosphate buffer (pH 7.0), 0.2 mL 10 mM ascorbic acid, 0.1 mL 3% H_2_O_2_, and 0.2 mL crude enzyme extract.

CAT activity was determined spectrophotometrically by monitoring the decrease in absorbance at 240 nm. The reaction mixture contained 2.8 mL of sodium phosphate buffer (50 mM, pH 7.0), 100 *μ*L of 3% H_2_O_2_, and 100 *μ*L enzyme extract.

Activity of LOX (EC 1.13.11.12) was detected by the procedure described by Surrey [32]. One unit of LOX was defined as a decrease of 0.01 OD value in absorbance per minute. The results of APX, CAT, and LOX were expressed as U·g^−1^ FW.

### 2.7. Statistical Analysis

The data in the paper are based on three or more replicates in each experiment, and the experiments were repeated independently for three times and similar change pattern was observed. Statistical significance was tested by one-way analysis of variance (ANOVA) using IBM SPSS Statistics (SPSS version 20.0, Armonk, NY), and the results were expressed as the means ± SD. Least significant difference test was performed on all data following ANOVA tests to test for significant (*P* < 0.05 or *P* < 0.01) differences between treatments.

## 3. Results

### 3.1. H_2_S Alleviates Postharvest Senescence and Rotting of Kyoho Grape

**Figure 1:**
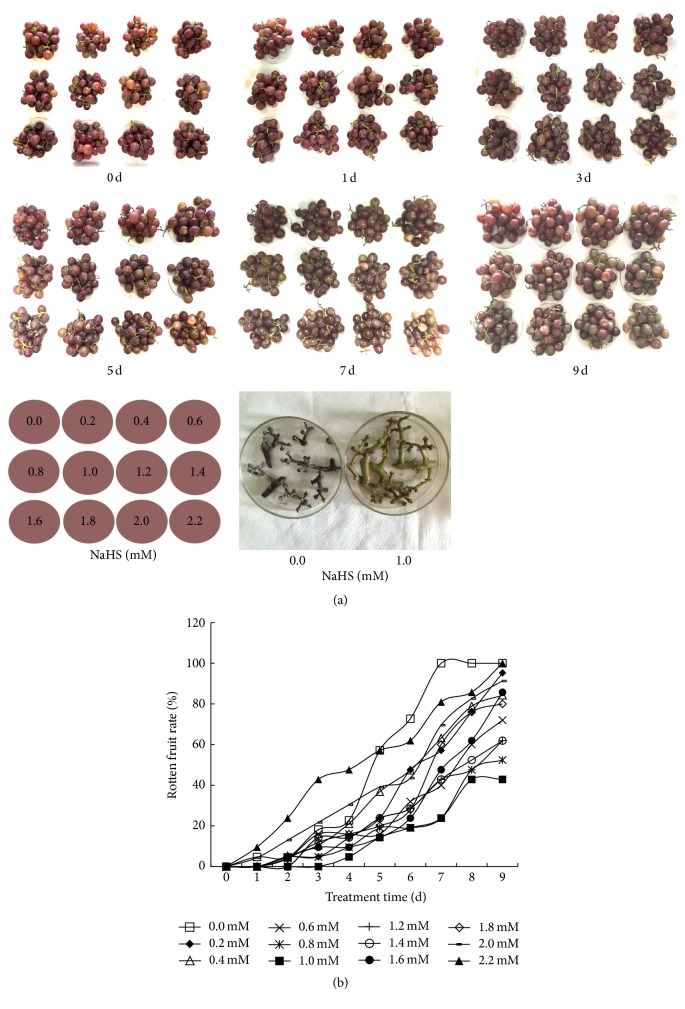
Hydrogen sulfide (H_2_S) treatment delays the senescence and rotting of Kyoho grapes in a dose-dependent manner. Grape clusters were fumigated with H_2_S released from different concentrations of aqueous NaHS (0, 0.2, 0.4, 0.6, 0.8, 1.0, 1.2, 1.4, 1.6, 1.8, 2.0, and 2.2 mM) and the photographs of grapes were taken every two days (a). Grape rachis of control and 1.0 mM NaHS treatment on day 5 of storage were presented in third panel of (a). Meanwhile, rotten fruit rates were recorded daily as shown in (b). The experiments and the following ones were carried out at 25°C and 85–90% relative humidity.

Grape clusters were fumigated with H_2_S released from aqueous solutions of NaHS ranging from 0.2 mM to 2.2 mM with water treatment as controls. The visual effects of H_2_S on delaying grape senescence, berry cracking, rotting, and threshing are shown in Figure 1(a). As for control berries and 2.2 mM NaHS treated ones, rotten fruit rate increased steadily with storage time, whereas 1.0 mM NaHS could remarkably decrease the rotten and threshing rate of grape berries and was used for subsequent experiments (Figure 1(b)).

### 3.2. Effect of H_2_S on the Browning Index of Grape Rachis, Weight Loss, Firmness, Total Soluble Solids, and Titratable Acidity of Berries

**Figure 2:**
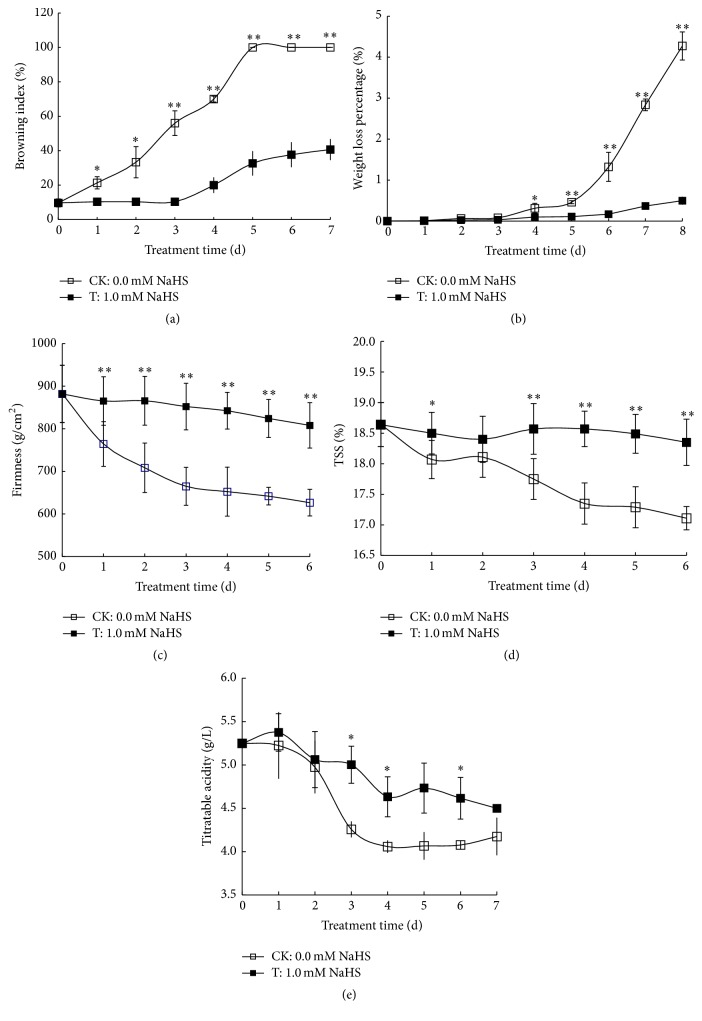
Effects of H_2_S on browning index of grape rachis (a), weight loss percentage of grape berries (b), grape berry firmness (c), total soluble solids (TSS) (d), and titratable acidity (e) in grape flesh. Grape clusters were fumigated with 1.0 mM H_2_S donor NaHS aqueous solution with water as the control groups for 0–8 d. Data are presented as means ± SD (standard deviation) (*n* = 3 rachis for (a), *n* = 3 grape clusters for (b), *n* = 8 grape berries for (c), *n* = 10 replicates for (d), and *n* = 3 replicates for (e)). The symbols *∗* and *∗∗* in this figure and the following ones stand for a significant difference between water control and 1.0 mM NaHS treatment at *P* < 0.05 and *P* < 0.01, respectively. FW = fresh weight.

Rachis browning is a common problem that affects grape quality and consumer preference. As shown in the lower right part of Figure 1(a), grape rachis in 1.0 mM NaHS treated grape clusters still retained a green appearance on day 5 of storage, while the control rachis developed serious browning. Also illustrated in Figure 2(a), browning index of water control rachis increased steadily to 100% on day 5 compared with 30% of NaHS treated ones.

Whole-cluster weight loss of H_2_S treatment and water control is presented in Figure 2(b). The weight loss percentage of control grape clusters went up steadily to about 4.3 on day 8 of storage, while the weight loss was effectively alleviated in H_2_S treatment. Berry firmness of water control declined gradually during storage, whereas only slight decrease in firmness was observed in berries treated with H_2_S (Figure 2(c)).

As shown in Figure 2(d), the content of soluble solids of control grape berries decreased sharply along with storage. However, H_2_S application maintained significantly higher levels of soluble solids compared with that of control except on day 2 of storage. Titratable acidity (Figure 2(e)) in control berries dropped sharply until day 4 and thereafter maintained a stable level, which is a symbol of an enhanced ripening. In contrast, titratable acidity in NaHS treatment showed a slower downward trend and was significantly higher than that of water control on days 3, 4, and 6 (Figure 2(e)).

### 3.3. Effect of H_2_S on the Contents of Chlorophyll and Carotenoid in Postharvest Grape Rachis and Pulp

**Figure 3:**
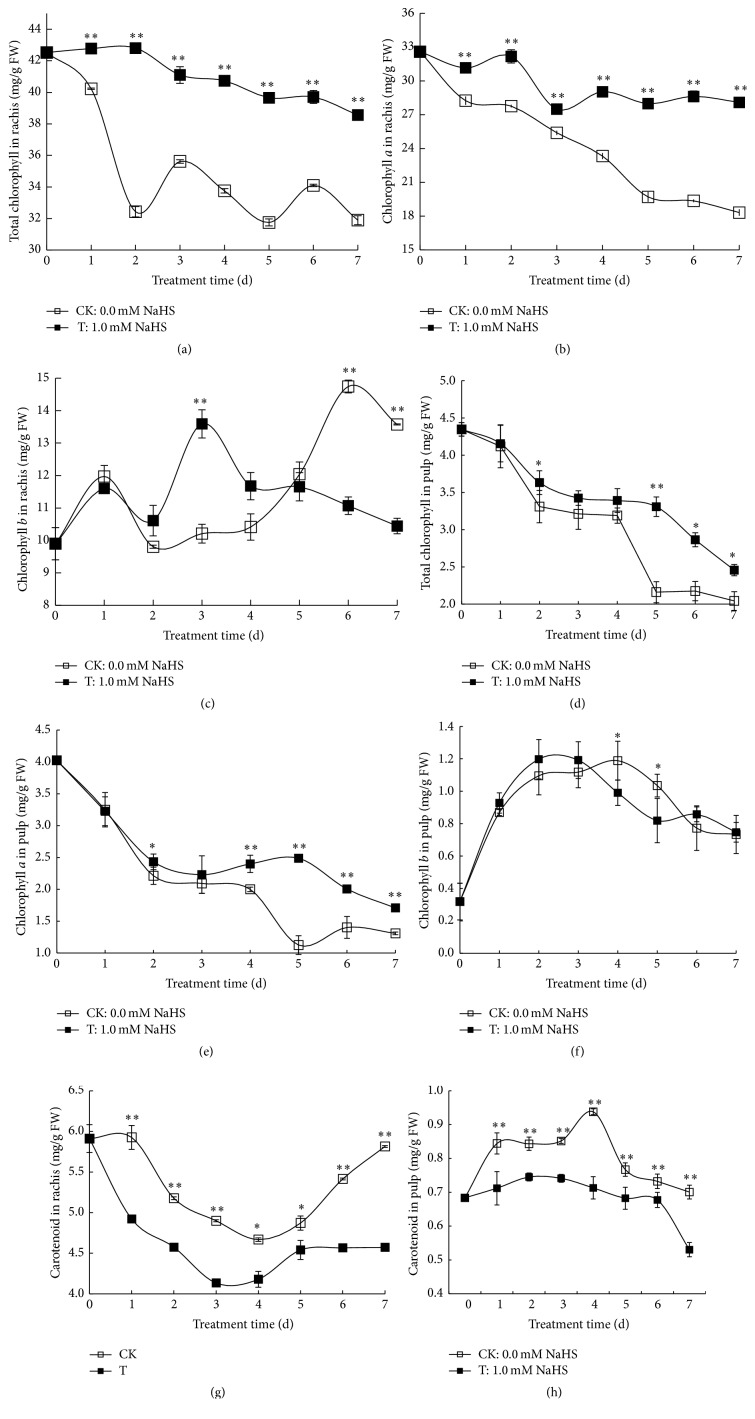
Effects of H_2_S on the contents of total chlorophyll (a) and chlorophyll* a* (b) and chlorophyll* b* (c) in rachis and total chlorophyll (d) and chlorophyll* a* (e) and chlorophyll* b* (f) in grape pulp and on the content of carotenoid in rachis (g) and grape pulp (h). Grape clusters were fumigated with 1.0 mM H_2_S donor NaHS aqueous solution with water as the control groups for 0–7 d. Data are presented as means ± SD (*n* = 3 for (a), (b), (c), and (g), *n* = 6 for (d), (e), (f), and (h)). FW = fresh weight.

Chlorophyll breakdown is shown to be associated with the first steps of the senescence process [33]. Besides, fruit ripening is often accompanied with the destruction of the green chlorophyll pigments and accumulation of yellow carotenoids in the flesh [34]. Thus, to understand how H_2_S alleviated rachis browning and berry senescence, we determined the contents of chlorophyll and carotenoid in rachis and pulp. Chlorophyll contents (Figures 3(a) and 3(d)) were expressed as the sum of chlorophyll* a* (Figures 3(b) and 3(e)) and chlorophyll* b* (Figures 3(c) and 3(f)). In rachis, total chlorophyll content as well as the amounts of chlorophyll *a* showed a decline trend during storage in both water controls and H_2_S treatment, while H_2_S fumigation maintained a relatively stable level of total chlorophyll and chlorophyll *a* during the storage (Figures 3(a) and 3(b)). Similarly, a decrease in total chlorophyll content in pulp was also observed in water controls and H_2_S treatment, whereas H_2_S helped to maintain significantly higher level of total chlorophyll on days 2, 5, 6, and 7 compared with water control (Figure 3(d)). The content of chlorophyll *a* in both control and H_2_S treatment showed a decreasing trend along with time, while the content in H_2_S treatment was significantly higher than that of control on days 2, 4, 5, 6, and 7 (Figure 3(e)). However, a slightly higher level of chlorophyll *b* was found in control grape compared with H_2_S-treated ones on days 4 and 5 (Figure 3(f)).

Changes of carotenoid content in rachis and pulp are shown in Figures 3(g) and 3(h). During the whole storage period, carotenoid content in control group was always higher than that of H_2_S treatment in both the rachis and pulp. In grape rachis, carotenoid content decreased and bottomed on day 4 for water control and day 3 for NaHS treatment followed by an increase (Figure 3(g)). Carotenoid content in grape pulp of water control increased steadily and peaked on day 4 followed by a decline, while only slight change was observed in H_2_S-treated berries except a drop on day 7. The data of chlorophyll and carotenoid content suggested the role of H_2_S in preventing chlorophyll breakdown and carotenoid accumulation in both grape rachis and pulp.

### 3.4. Effect of H_2_S on the Contents of Ascorbic Acid, Flavonoid, Total Phenolics, Reducing Sugar, and Soluble Protein in Grape Pulp

**Figure 4:**
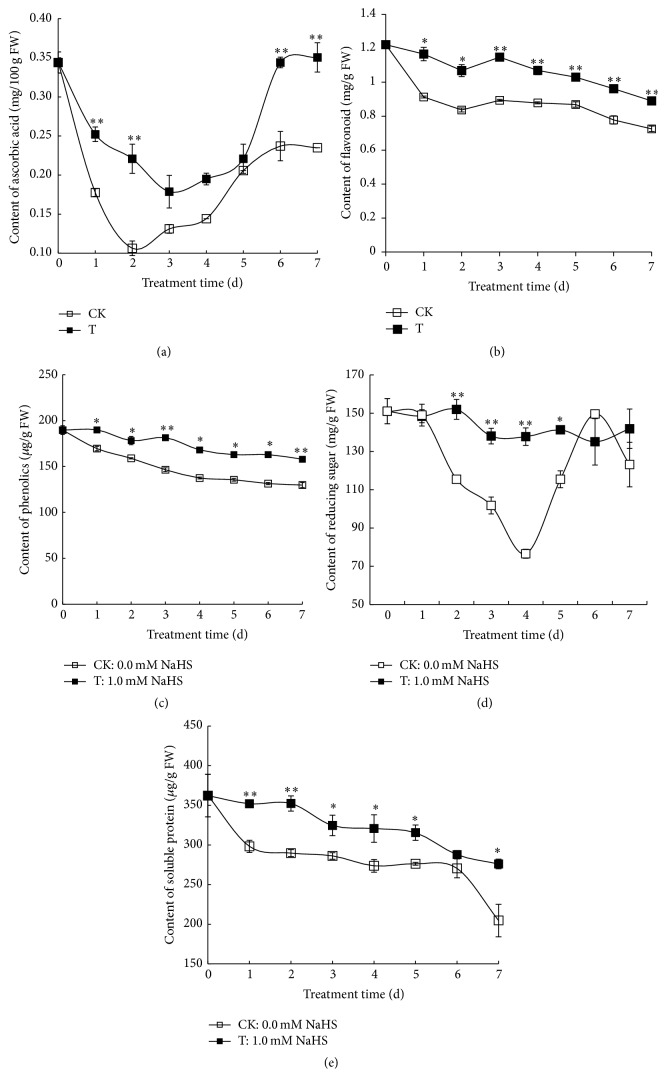
Effects of H_2_S on the contents of ascorbic acid (a), flavonoid (b), total phenolics (c), reducing sugar (d), and soluble protein (e) in grape pulp. Grape clusters were fumigated with 1.0 mM H_2_S donor NaHS aqueous solution with water as the control groups for 0–7 d. Data are presented as means ± SD (*n* = 3). FW = fresh weight.

Ascorbic acid, flavonoid, and phenols are natural antioxidants and important nutrient traits of fruit. As shown in Figure 4(a), the content of ascorbic acid decreased to a bottom on day 2 for control and on day 3 for H_2_S treatment followed by a gradual increase. However, H_2_S treatment sustained significantly higher content of ascorbic acid on days 1, 2, 6, and 7 in comparison to water control.  Figure 4(b) illustrated a decreasing trend of flavonoid content in grape berries treated with H_2_S or not, whereas H_2_S treatment sustained significantly higher level of flavonoid compared with water control. Similar decreasing trend was also observed in the changes of phenolics content (Figure 4(c)). However, in comparison to water control, H_2_S fumigation significantly alleviated the decrease and maintained higher content of phenolics during the whole storage.

The contents of reducing sugar and soluble protein in grape berries are shown in Figures 4(d) and 4(e). Reducing sugar, as a primary energy substance, is a key energy source in postharvest fruit and vegetables. Reducing sugar in water control declined sharply and bottomed on day 4 followed by a surge till day 6. However, there was only slight fluctuation of reducing sugar in H_2_S-treated berries and significantly higher level of reducing sugar was observed on days 2 to 5 relative to that of water control. Soluble protein content in both water control and H_2_S decreased continually during the storage, but H_2_S treatment significantly alleviated the decrease, suggesting the role of H_2_S in preventing protein degradation.

### 3.5. Effect of H_2_S on the Contents of MDA, H_2_O_2_, and O_2_^∙−^ in Grape Pulp

**Figure 5:**
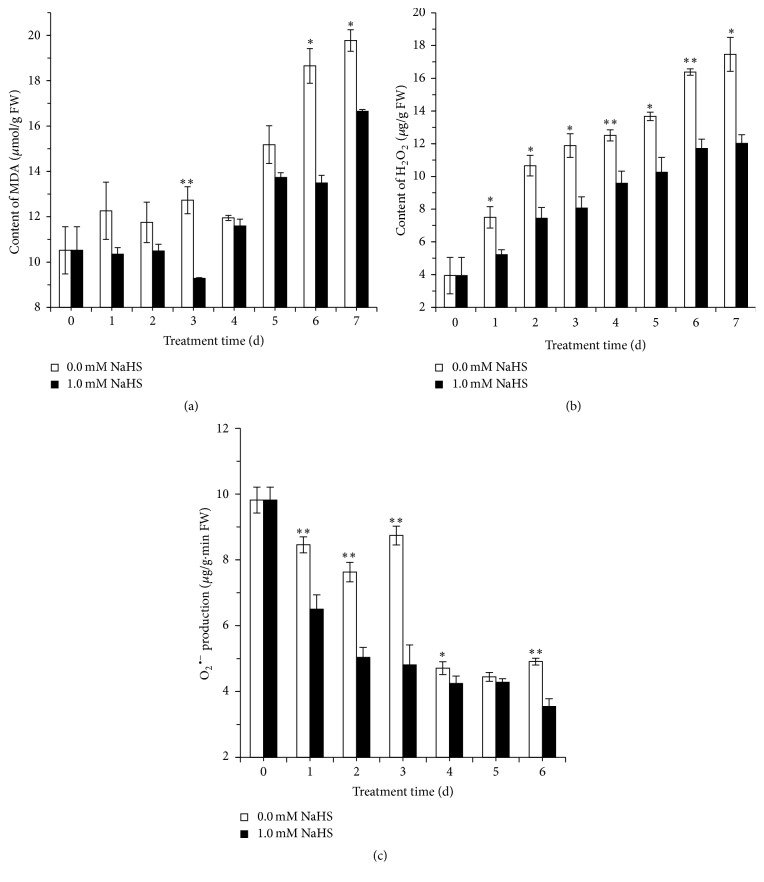
Effects of H_2_S on the contents of malondialdehyde (MDA) (a), hydrogen peroxide (H_2_O_2_) (b), and superoxide anion (O_2_^∙−^) (c) production rate in grape pulp. Grape clusters were fumigated with 1.0 mM H_2_S donor NaHS aqueous solution with water as the control groups for 0–7 d. Data are presented as means ± SD (*n* = 3). FW = fresh weight.

The contents of MDA and H_2_O_2_ and the generation of O_2_^∙−^ in grape fumigated with H_2_S or water are shown in Figure 5. MDA is determined as an index of lipid peroxidation. As shown in Figure 5(a), MDA content in water control pulp fluctuated during the first four days of storage followed by a surge. An increase of MDA content was also observed in H_2_S-treated berries on day 4, but H_2_S treatment significantly reduced MDA accumulation on days 3, 6, and 7, implicating the role of H_2_S in alleviating lipid peroxidation.

The overproduction of reactive oxygen species (ROS) is universally occurring during fruit senescence [21, 22].  Figure 5(b) shows that H_2_O_2_ content in grape pulp increased steadily in both H_2_S treatment and water control, while H_2_S treatment significantly reduced H_2_O_2_ accumulation. However, the content of O_2_^∙−^ in control pulp fluctuated during the first 3 days of storage followed by a decrease on day 4. In contrast, the content of O_2_^∙−^ in H_2_S treatment declined continuously in pulp and was significantly lower compared to water control except on day 5.

### 3.6. Effect of H_2_S on the Activities of APX, CAT, and LOX in Grape Peels and Pulp

**Figure 6:**
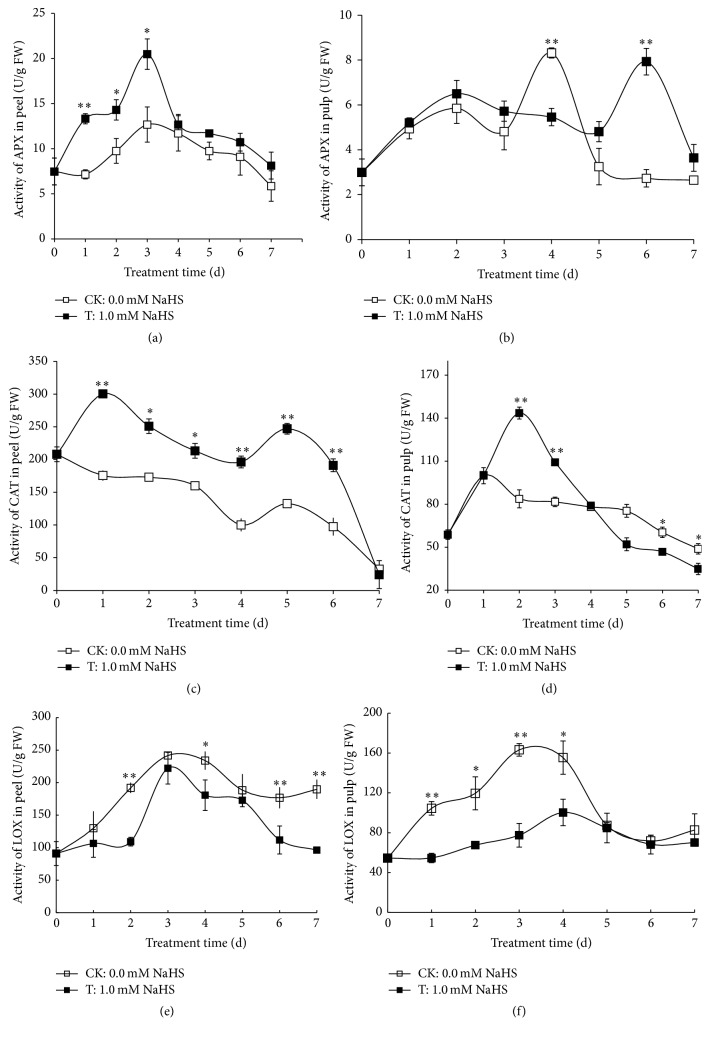
Effects of H_2_S on the activities of ascorbate peroxidase (APX) in grape peels (a) and pulp (b), catalase (CAT) in peels (c) and pulp (d), and lipoxygenase (LOX) in peels (e) and pulp (f). Grape clusters were fumigated with 1.0 mM H_2_S donor NaHS aqueous solution with water as the control groups for 0–7 d. Data are presented as means ± SD (*n* = 3). FW = fresh weight.

To further understand the role of H_2_S in ROS metabolism in grape, we determined the activities of enzymes involved in oxidative metabolism in plants, such as APX, CAT, and LOX. As showed in Figure 6(a), the activity of APX in grape peels of both control and H_2_S treatment increased steadily and peaked on day 3 of storage followed by a gradual decline. However, APX activity in H_2_S-treated peels was significantly enhanced on days 1 to 3 compared with that of water control. The changes in APX activity in grape pulp are shown in Figure 6(b). APX activity in control peels increased during the first 2 days of storage and then fluctuated and peaked on day 4 followed by a drop on day 5 and then a plateau. Similar increase in APX activity was observed in H_2_S-treated peels till day 2 followed by a slight decrease till day 5. However, H_2_S treatment induced an about 3-fold increase in APX activity on day 6 in grape peels compared with control. As shown in Figure 6(c), H_2_S treatment induced a swift increase of CAT activity on day 1 in grape peels followed by a gradual decrease till day 4. Then, an increase was observed in CAT activity in peels treated with H_2_S followed by a drop. Similar trend of the changes in CAT activity was seen in control peels, except that there was no activity increase on day 2 compared with that of H_2_S treatment (Figure 6(c)). However, H_2_S significantly promoted CAT activity in peels during the whole storage in comparison to water control except on day 7.  Figure 6(d) illustrates the effect of H_2_S on CAT activity in grape pulp. CAT activity in control pulp increased on day 1 followed by a slight decrease thereafter, whereas H_2_S was found to induce an increase during the first two days of storage followed by a gradual increase. CAT activity in H_2_S-treated pulp was significantly higher than that of control on days 2 and 3 but was lower than that of control on days 6 and 7.

LOXs are enzymes that catalyze the hydroperoxidation of polyunsaturated fatty acids. As shown in Figure 6(e), LOX activity in control peels increased steadily and reached a maximum value on day 3 followed by a slight decline. Similar trend of changes of LOX activity was observed in H_2_S-treated peels, while H_2_S significantly reduced LOX activity on days 2, 4, 6, and 7 compared with water control.  Figure 6(f) showed that H_2_S also attenuated LOX activity in pulp during the first 4 days of storage. LOX activity in control pulp rose significantly till day 3 followed by a drop on day 5. However, an attenuated increase in LOX activity was observed in H_2_S-treated pulp during the first 4 days of storage (Figure 6(f)).

## 4. Discussion

Table grapes are highly perishable and their quality deteriorates quickly after harvest because of water loss and fungal spoilage especially in developing countries where cold chain transportation is not always available [1]. Besides, rachis browning also has a great impact on consumer preference. Here, we provide an alternative strategy other than SO_2_ fumigation to maintain the freshness of grape berries and green color of the rachis. Water loss is responsible for large and significant changes in the composition and metabolism of detached fruit, which induces changes in color and palatability and loss of nutritional quality [35, 36]. We found that H_2_S treatment effectively reduced weight loss in grape clusters and maintained higher berry firmness compared to water control (Figures 2(b) and 2(c)). Higher titratable acidity (TA) indicates a marked delay in process of maturation and ripening and loss of acidity can cause the fruits to taste insipid during storage. H_2_S fumigation alleviated the decrease in TA during grape storage, further suggesting the role of H_2_S in delaying fruit maturation and ripening (Figure 2(e)).

During fruit ripening and senescence, green chlorophyll pigments were decomposed and yellow carotenoids accumulated in the flesh [33, 34]. In the present research, chlorophyll degradation was observed in both grape rachis and pulp, which was consistent with previous findings that during storage there was upregulation of chlorophyll breakdown-related genes in rachis [37]. However, H_2_S significantly prevented chlorophyll degradation and carotenoid accumulation in both rachis and pulp, further confirming the antisenescence role of H_2_S in plants (Figure 3). Although grapes are nonclimacteric, the effect of ethylene on ripening at veraison is well established [38]. Rachis browning was believed to be associated mainly with dehydration, but there is evidence showing that ethylene acts as a major factor in rachis browning [1, 39]. Recent study shows that 1-methylcyclopropane (1-MCP), which is a potent inhibitor of ethylene action, delays rachis browning in three table-grape varieties whereas ethylene tends to enhance it [39]. Besides, treatment with cytokinin or abscisic acid (ABA) improves rachis quality during storage, further suggesting the involvement of senescence during rachis browning because cytokinin and ABA are known to have antisenescent effect in plant [40, 41]. Thus, our finding of the role of H_2_S in alleviating grape senescence and rachis browning highlights the possibility that H_2_S might act as an antagonist to counteract ethylene-induced fruit senescence.

Plant senescence is usually accompanied with the accumulation of ROS which can potentially cause oxidative damage to cellular components, including lipid, protein, and nucleic acid [42]. The metabolism of ROS is controlled by a series of antioxidant enzymes including CAT and APX. We found that H_2_O_2_ was accumulated in control grape pulp while H_2_S effectively reduced H_2_O_2_ accumulation and O_2_^∙−^ content (Figures 5(b) and 5(c)). APX and CAT are the two enzymes responsible for H_2_O_2_ breakdown. In the present study, H_2_S treatment significantly enhanced the activities of APX and CAT in both grape peels and pulp during storage, which helped to scavenge excessive ROS and reduced ROS-caused damage to tissues (Figures 6(a), 6(b), 6(c), and 6(d)). In addition to the antioxidative effect of antioxidant enzymes, nonenzymatic antioxidants or nutritional components such as ascorbic acid, flavonoid, and phenolics, which are important quality parameters used to evaluate the storage effect on table grapes, also help to maintain a balanced ROS metabolism by quenching ROS [43]. In the present research, H_2_S was found to maintain higher levels of ascorbic acid, flavonoid, and phenolics in grape pulp compared with water control (Figures 4(a), 4(b), and 4(c)), highlighting the positive role of H_2_S in grape storage. Other compounds such as chitosan-glucose complex, which has superior antioxidant activity in grape, were also found to delay the declines of ascorbic acid and titratable acidity and to induce antioxidant enzymes, thereby extending the postharvest life of grape [44]. Further, preharvest polyamines application which maintained higher value of antioxidant activity during grape storage also improved grape quality as indicated by the higher levels of phenolics and anthocyanins and alleviated weight loss and softening [45]. All the above publications highlighted the central role of oxidative stress during grape senescence and the effectiveness of antioxidant compounds (including H_2_S, polyamines, and chitosan-glucose complex) in delaying grape senescence.

LOX, as one of the key enzymes in membrane lipid peroxidation, is capable of catalyzing the peroxidation of unsaturated fatty acids to form a series of reactive oxygen species and thereby causing disorders in the normal physiological metabolic activity of cells [46]. We found that H_2_S significantly inhibited the increase in LOX activity and meanwhile reduced the accumulation of MDA, which is a product of lipid peroxidation and a marker of oxidation of the plasma membrane [46] (Figures 5(a), 6(e), and 6(f)).

In all, our results indicated that H_2_S could alleviate postharvest senescence of grape and maintain high fruit quality by decreasing ROS accumulation, improving antioxidant enzyme activities, and reducing lipid peroxidation, thereby maintaining the stability of the membrane structure.

## 5. Conclusion

In summary, we demonstrated that exogenous application of H_2_S effectively alleviated postharvest senescence of grapes by preventing rachis browning and berry rotting and maintaining grape firmness, soluble solids, titratable acidity, and natural antioxidants during postharvest storage. The protective role of H_2_S in grapes could be attributed to the increased activities of ROS-scavenging enzymes which bring about a repression on the production of ROS such as H_2_O_2_ and O_2_^∙−^ and to the decreased level of LOX activity. In all, we provided strong evidence that H_2_S effectively alleviated postharvest senescence and rotting of Kyoho grape by modulating antioxidant enzymes and attenuating lipid peroxidation. Considering the critical role of ethylene in postharvest senescence of grape berries and rachis, it will be interesting to know whether H_2_S is antagonistically involved in ethylene pathway.
